# Long-Term Follow-Up of the Auditory Threshold After a Fully Implantable Middle Ear Implant

**DOI:** 10.3389/fneur.2022.834402

**Published:** 2022-02-10

**Authors:** Simonetta Monini, Chiara Filippi, Gerardo Salerno, Maurizio Barbara

**Affiliations:** Otorhinolaryngology Clinic, NESMOS Department, Sapienza University, Rome, Italy

**Keywords:** active middle ear implant, sensorineural hearing loss (SNHL), fully implantable, auditory rehabilitation, bone conduction threshold change

## Abstract

A fully implantable active middle ear device has been proposed and indicated for the rehabilitation of bilateral moderate or moderate-to-severe sensorineural hearing loss, assuming it would overcome the disadvantages of a conventional hearing aid. The indications have further been extended to severe or severe-to-profound forms of hearing loss in the case of an expected limited or null efficacy of hearing aids. While the literature has highlighted several positive aspects of the device, including a better quality of life related to its invisibility, the improvement of auditory and perceptual functions has not been controlled for throughout a long period of follow-up. The present study aimed to verify the behavior of the auditory threshold, especially the bone conduction (BC) component, in the implanted ear in a group of implantees affected by initial bilateral symmetric hearing loss of different severity grades. The BC threshold was assessed preoperatively at activation and at the last follow-up (ranging from 4 to 12 years) in the implanted ear, and preoperatively and at the last follow-up in the contralateral ear, to monitor eventual deteriorated values in both ears over time. The pure tone average (PTA; 250–4,000 Hz), speech reception threshold (SRT) and the maximum word recognition score as a percentage (% WRS) and in dB HL were measured in the implanted ear to verify the efficacy of the device after the first fitting at device activation. A significant worsening of the BC threshold with respect to the baseline threshold was noticed during further follow-up. When comparing the implanted ear with the contralateral ear, a significant worsening of the bone PTA was assessed in the former with respect to the contralateral ear. Despite the worsened hearing found in the implanted ears, the beneficial gains in PTA and speech audiometry observed at the first activation remained constant at the follow-up, thus showing an extension of the efficacy of this device in aiding those with up to the most severe forms of sensorineural hearing loss.

## Introduction

Active middle ear implants (AMEIs), either semi- or fully implantable, have been proposed as an alternative to conventional hearing aids (CHA) in subjects with moderate-to-severe hearing loss (HL) to overcome some of the issues observed with CHA, such as limited high-frequency amplification and incompatibility with a chronic inflammatory condition of the external auditory canal (1). One fully implantable AMEI (FI-AMEI) has also been proposed for subjects with a greater degree of sensorineural hearing loss (SNHL), such as the severe or severe-to-profound type for which CHA would be undoubtedly inadequate (2–4). In this regard, an 11–13 dB difference was reported when comparing the hearing performances of the same subject using the FI-AMEI with that of using a last generation CHA (5), a difference theoretically related to the FI-AMEI's better high-frequency amplification. A similar observation has also been confirmed by specific questionnaires on the benefits and perceived quality of sound (6).

After the FI-AMEI application, the implantees' auditory conditions displayed some modifications that are not exclusively related to the device function. In fact, all the subjects showed a deterioration of the air-conduction thresholds that shifted the purely sensorineural into a mixed type of hearing loss. This finding is due to the interruption of the incudo-stapedial continuity by the partial removal of the incus long process, a necessary surgical step that allows for the minimization or elimination of possible distorted phenomena due to the piezoelectric bridge between the incus-cemented microphonic sensor and the stapes-cemented driver (7). Considering that postoperative hearing improvement is achieved and measured via direct mechanical stimulation of the inner ear, this additional conductive component appears to have limited importance for device function, while it will negatively impact the eventual use of CHA in the operated ear. In addition, should the occurrence of eventual postoperative complications (middle ear fibrous growth or device failure, for example) prompt a device's explantation, ossicular reconstruction will become necessary.

Conversely, the eventual deterioration of the bone conduction (BC) threshold should be considered to be an undesirable outcome that may occur just after surgery, analogous to other ear surgical procedures (stapedotomy, tympanoplasty, etc.). When the BC decrease is of limited extent (<10 dB), it does not usually impact the functional output that is regulated at the first activation fitting. Conversely, a sensible loss of BC threshold may affect device function, as may be observed with any hearing rehabilitative tool, ultimately requiring alternative solutions (8). The likelihood of BC deterioration after the present FI-AMEI use was previously reported in an FDA phase 2 clinical trial, which accounted for only 1 year of use (9).

The aim of the present study was to obtain evidence of eventual BC threshold deterioration in our FI-AMEI cohort, who were followed up for a long period of time, and to shed some light on possible correlations with some parameters, such as the initial degree of SNHL and the shape of the audiogram. In addition, we investigated the role played by SNHL etiology in a select cohort of implantees by comparing the implanted ear with the contralateral, non-implanted ear, when both were affected by the same initial degree of hearing loss. Finally, we investigated the possibility of either a persistent beneficial effect of the FI-AMEI despite BC threshold deterioration or the need for transitional rehabilitation by a cochlear implant.

## Materials and Methods

A retrospective, non-randomized and self-controlled study was performed, taking into consideration, in each subject, the comparison between the implanted and contralateral ears. The Esteem® (Envoy Medical, St. Paul, USA) is the first and, presently, the only fully implantable active middle ear implant (FI-AMEI) approved by the Food and Drug Administration (10) for the rehabilitation of moderate and moderate-to-severe SNHL. Between 2007 and 2019, 43 patients received the Esteem FI-AMEI at a single implanting center. All of them were selected on the basis of FDA-approved manufacturer recommendations that included moderate or moderate-to-severe bilateral sensorineural hearing loss, with a type A tympanogram and at least 40%-word discrimination (7). In all patients, the implant was activated 6 weeks after surgery, with the aided auditory function assessed by pure tone average (250–4,000 Hz), speech reception threshold (SRT), maximum word recognition score (WRS) as a percentage and in dB HL. The unaided threshold was also assessed at the time of activation to quantify the degree of conductive loss that was due to ossicular chain interruption as well as the level of the BC threshold. Each patient was scheduled for an annual control of the same auditory parameters in both the implanted and contralateral ears. During these periodic controls, ranging from 4 to 12 years after surgery, in case of a documented change of the auditory threshold or referred decrease of device performance, and when battery replacement was needed, the patients underwent a new regulation of the device on the basis of the newly assessed BC threshold as well as on Envoygram® data (Envoy Medical, St Paul, MN, USA), with adjustment of amplification levels when needed. In fact, when an eventually lowered BC threshold was not measurable due to the technical limitations of a standard audiometer, the *in-situ* BC measurement was carried out by specific software provided by the manufacturer (Envoygram), which allows for the detection of lower (worse) levels of the BC threshold. Only patients with symmetric baseline SNHL (*n* = 20) so as presenting a mean threshold difference between the two ears <15 dB (11) were included in the study group.

The absolute and mean values of baseline and last follow-up BC thresholds (250–4,000 Hz) in the ipsilateral and contralateral ears were assessed and compared. The auditory and perceptual gains at the last follow-up were assessed by PTA and speech audiometry and compared with the baseline fitting (activation) values to confirm the efficacy of the device. Visual Analog Scale (VAS) was also administered for the subjective evaluation of audiological benefit at 1 year after implant activation and at follow-up (4–12 years) to assess if the eventual bone threshold deterioration could have impacted on patients' satisfaction.

Among the study group, subjects with moderate-to-severe HL (Group A, *n* = 13) were distinguished from those with a severe-to-profound HL (Group B, *n* = 7). The eventual influences of follow-up time and degree of BC worsening at the follow-up between the two subgroups were calculated after quantifying the statistical significance of the eventual deterioration in each subgroup.

### Statistical Analysis

In the implanted and contralateral ears, the difference between the baseline and last follow-up BC thresholds for each 250–4,000 Hz PTA and for the global means was assessed. In each patient, the mean BC PTA worsening at the last follow-up with respect to the preoperative values for the range 250–4,000 Hz was compared using Student's *t*-test in the implanted and contralateral ears. In the implanted ear, SRT, percent WRS and WRS dB HL values were compared by Student's *t*-test at the following times: preoperatively vs. activation, activation vs. last follow-up and last follow-up vs. preoperatively. The student's *t*-test for small samples was also used to compare subgroups A and B for follow-up timing and to assess the difference between them in BC worsening at the time of follow-up with respect to the baseline threshold. In both ears, the correlation between the follow-up time and the degree of hearing loss, as well as the correlation between the follow-up hearing loss degree and the preoperative value, were investigated. Data were expressed as the mean and standard deviation. The differences between the two ears were expressed as differences between the means and 95% CI. The correlation between two variables was evaluated using the Pearson correlation coefficient. The significance indication was fixed with a *p* < 0.05.

## Results

Out of the 43 subjects who received the FI-AMEI at our tertiary care center, 9 were lost to follow-up (living abroad or followed by other centers); 1 patient was explanted after 6 years of continuous use for other medical problems (liver transplant program) and 2 patients had the implant removed due to post-operative complications (implant reject, malfunctioning). One patient was excluded because of unilateral hearing loss.

Twenty of the 30 remaining patients (6 females, 14 males, aged 21–70 years, mean age 43 years) presenting preoperatively with symmetric sensorineural hearing loss and BC worsening at the follow-up, were the object of the study. Because none of these subjects showed BC worsening prior to 4 years post-surgery, this time limit was taken as the initial time (T0) for the follow-up, while the other time intervals were ≥5 years, between 5 and 10 years and >10 years.

The baseline hearing threshold allowed us to distinguish moderate-to-severe and severe-to-profound hearing loss, which formed the basis of the two groups according to more (B) or less (A) severe hearing loss. A further distinction was made regarding the shape of the threshold that was identified as either flat or down-sloping.

At the baseline evaluation, hearing loss was mild-to-moderate in one subject, moderate in 15 subjects, and moderate-to-severe in 4 subjects. The shape of the threshold curve was down-sloping in 5 subjects and flat in the remaining 15. A similar shape of the baseline threshold curve was found in the contralateral ear. All the operated ears showed a mean conductive type of loss of 35 dB (250–4,000 Hz). The minimum timing of the follow-up was 4 years, with a maximum of 12 years (mean 7.45 years). The follow-up time was <5 years in 7 subjects; >5 <10 years in 8 subjects; >10 <12 years in 5 subjects. At the last follow-up, the implanted ears showed moderate-to-severe HL in 10 subjects and severe-to-profound HL in 10 subjects; the contralateral ear showed mild-to-moderate HL in 1 subject, moderate HL in 14 subjects and moderate-to-severe HL in 5 subjects.

### Implanted Ear

The mean preoperative and postoperative BC threshold levels in the implanted ear were 56.1 dB (min 38, max 71), with partial and total values of the BC threshold shown in Table 1. The difference between the mean BC PTA at the last follow-up and the preoperative value was 18.6 dB, with a confidence interval of 15.62–21.58 and a significance level indicated by the *p*-value for the mean PTA as well as for each frequency (*p* < 0.0001) (Table 1).

**Table 1:** Mean (250–4,000 Hz) pre-operative and follow-up bone conduction threshold levels in the implanted and contralateral ear.

**Variable**	**Esteem preop-postop *n* = 20**	**Esteem follow up *n* = 20**	**Difference (95% CI)**	***P*-value**	**CTR preop *n* = 20**	**CTR follow up *n* = 20**	**Difference (95% CI)**	***P*-value**
Age (SD) (yr)	42.65 (13.33)	–	–	–	42.65 (13.33)	–	–	–
Sex % female	30.00	–	–	–	30.00	–	–	–
BC at 250 Hz (SD) (dB)	34.50 (12.97)	62.50 (18.09)	27.75 (22.04–33.45)	**<0.0001**	34.75 (13.81)	40.50 (12.66)	5.75 (0.93–10.57)	**0.0220**
BC at 500 Hz (SD) (dB)	48.00 (11.05)	71.25 (10.11)	23.25 (19.75–26.75)	**<0.0001**	49.25 (15.41)	56.50 (13.87)	7.25 (3.13–11.37)	**0.0016**
BC at 1,000 Hz (SD) (dB)	62.00 (11.85)	75.00 (9.31)	13.00 (9.25–16.75)	**<0.0001**	62.25 (13.32)	68.50 (10.53)	6.25 (2.86–9.63)	**0.0010**
BC at 2,000 kHz (SD) (dB)	66.75 (11.72)	82.50 (8.66)	15.75 (11.93–19.57)	**<0.0001**	67.50 (16.26)	72.25 (10.94)	4.75(0.29–9.21)	**0.0380**
BC at 4,000 Hz (SD) (dB)	69.25 (11.27)	82.50 (10.70)	13.25 (8.49–18.01)	**<0.0001**	69.25 (18.01)	75.00 (12.67)	5.75 (0.42–11.07)	**0.0360**
BC at 250–4,000 Hz (SD) (dB)	56.10 (8.69)	74.70 (7.90)	18.60 (15.62–21.58)	**<0.0001**	56.60 (11.98)	62.55 (9.58)	5.95 (0.7–14.1)	**0.0018**

*BC, Bone Conduction; CTR, Contralateral ear. Bold values indicate the statistically-significant data*.

**Figure 1:**
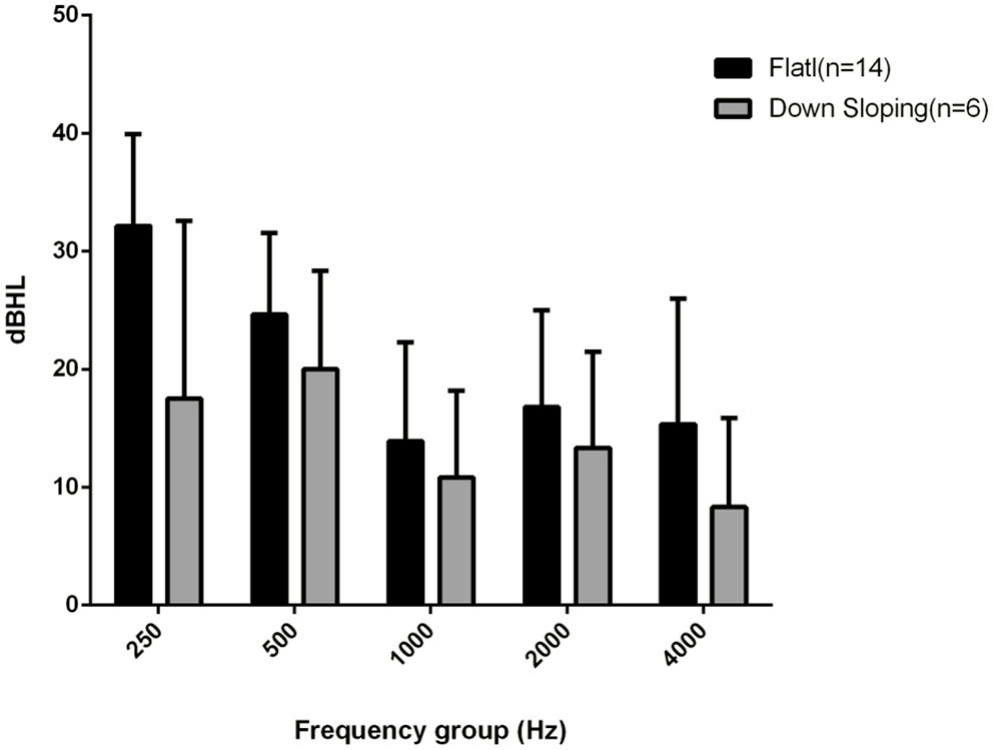
Bone conduction threshold levels at follow-up evaluation in down-sloping (n. 6) and flat-type curves (n. 14).

The mean BC threshold deterioration was 14 dB HL in the down-sloping curves and 21 dB HL in the flat-type curves (Figure 1). The worsening was more evident at 250 and 500 Hz in both groups; at the high frequency level, the down-sloping curves showed less deterioration than that of the flat-type curves.

### Contralateral Ear

The mean BC threshold level was 56.6 at the baseline and 62.55 at the last follow-up. The difference between the mean BC PTA at the last follow-up and the baseline threshold was 5.95 dB (confidence interval of 0.7–14.10, p for mean = 0.018, p for each frequency range 250–4,000 Hz < 0.05). The partial and total values of the baseline and last follow-up BC thresholds are shown in Table 1.

**Figure 2:**
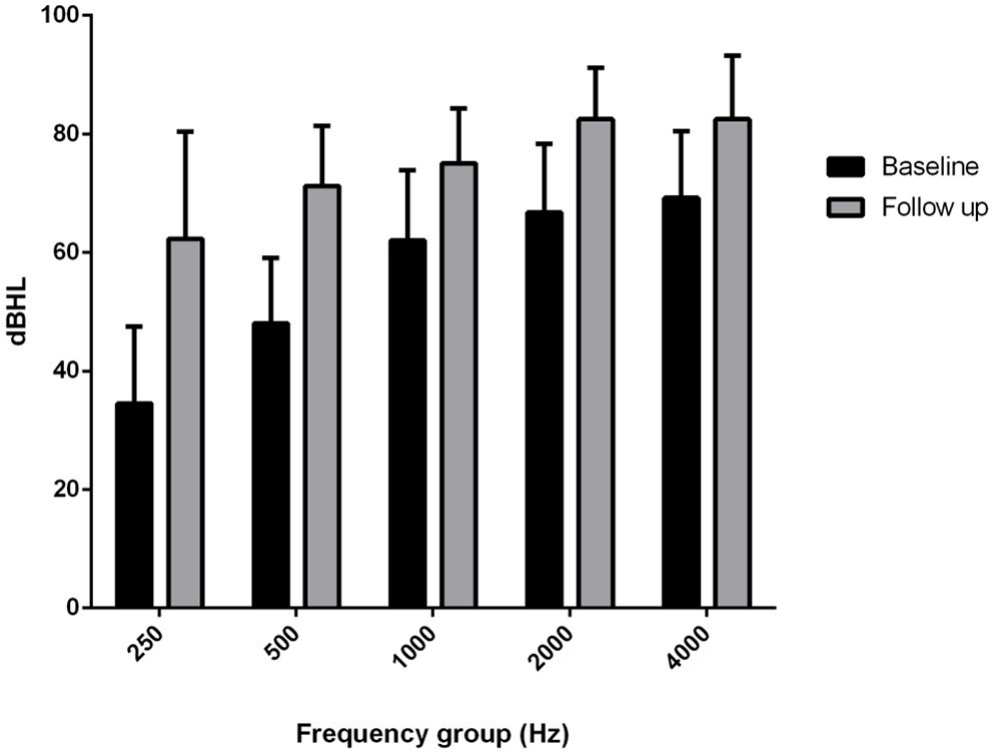
Bone conduction threshold levels in the Esteem-implanted ear: baseline vs. follow-up (4–12 years).

**Figure 3:**
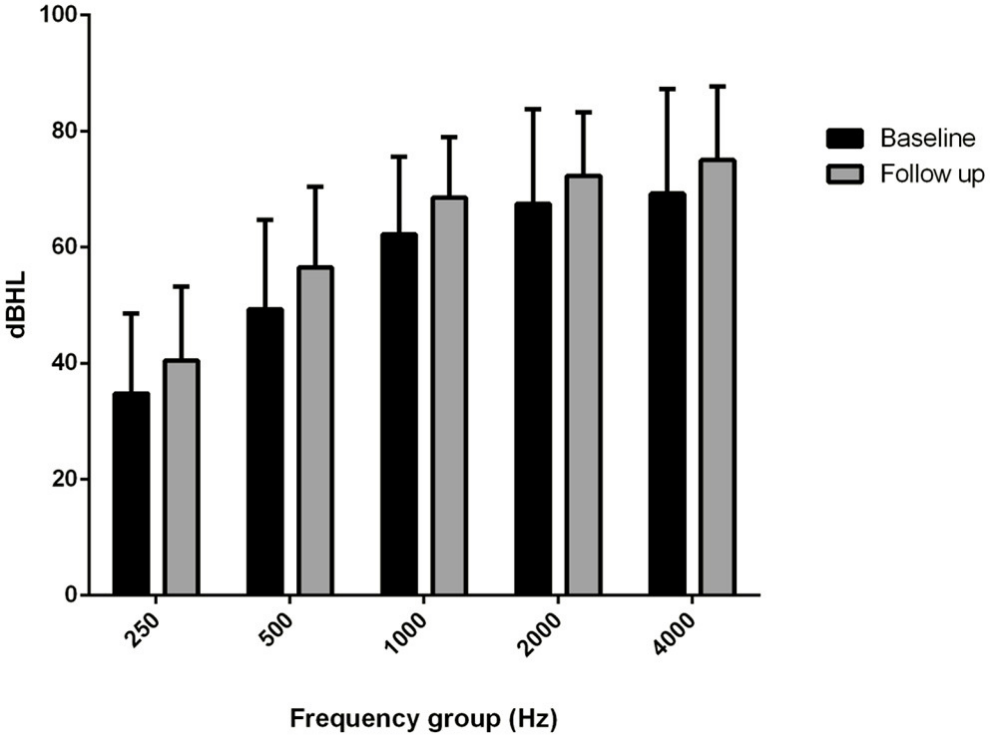
Bone conduction threshold levels in the contralateral, non-implanted ear: baseline vs. follow-up (4–12 years).

The comparison of the BC PTA value at the last follow-up with that of the baseline situation in the implanted and in the contralateral ear showed a mean worsening in the implanted ear of 18.6 dB compared with 6 dB in the contralateral ear, with a difference of 12.5 dB (Figures 2, 3), and with a more significant worsening of the AMEI ear with respect to the contralateral ear (*p* = 0.0000).

### FI-AMEI Efficacy at Activation and at the Last Follow-Up

**Figure 4:**
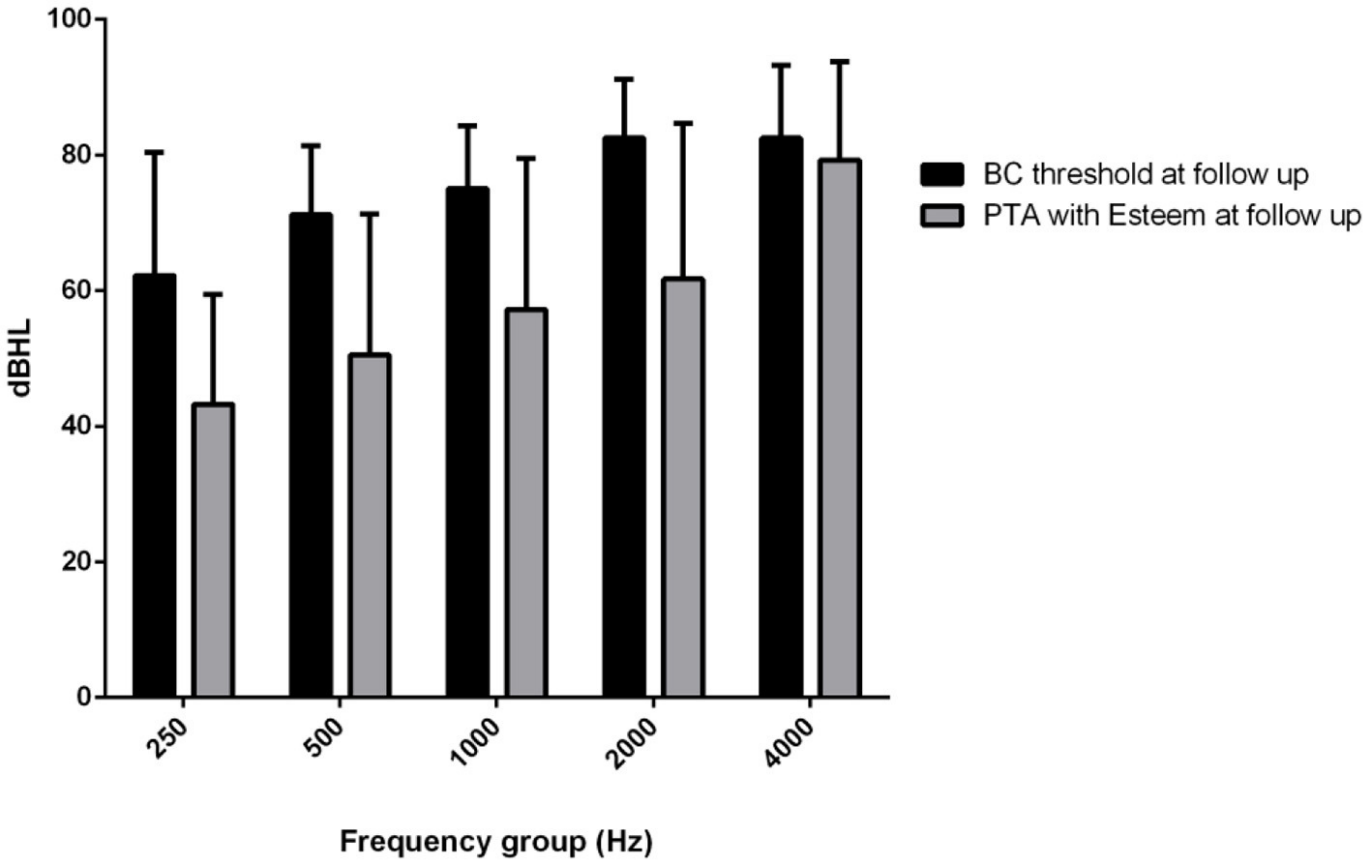
Mean (250–400 Hz) headphone PTA values and bone conduction threshold values in the Esteem-implanted ear at the follow-up evaluation.

**Figure 5:**
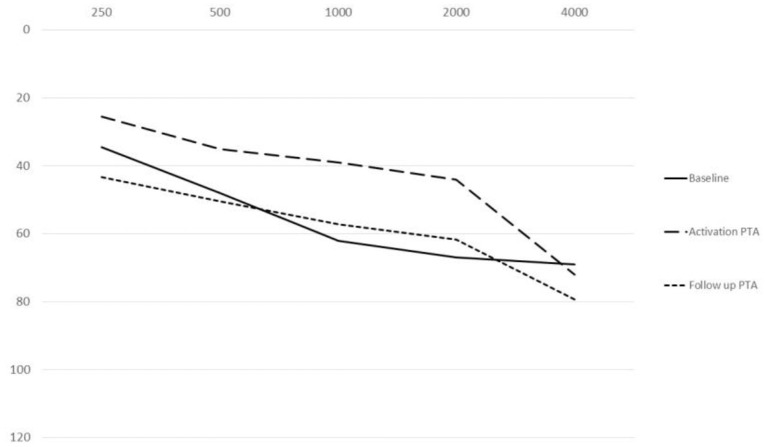
Comparison among PTA threshold values at baseline, activation and follow-up.

The mean PTA implant values at activation were 25.5 dB HL at 250 Hz, 35 dB HL at 500 Hz, 39 dB HL at 1,000 Hz, 44 dB HL at 2,000 Hz and 72 dB HL at 4,000 Hz. The mean PTA implant values at the last follow-up were 43.25 dB HL at 250 Hz, 50.5 dB HL at 500 Hz: 57.25 at 1,000 Hz, 61.75 dB HL at 2,000 Hz and 79.25 dB HL at 4,000 Hz. The mean PTA implant gain at activation with respect to the BC threshold was 13 dB HL, and at the last follow-up, with respect to the follow-up BC threshold at the different frequencies, it was 19 dB at 250 Hz, 20 dB at 500 Hz, 18 dB at 1,000 Hz, 21 dB at 2,000 Hz, and 3 dB at 4,000 Hz (Figure 4), with a mean gain of 16.2 dB HL (Figure 5).

**Figure 6:**
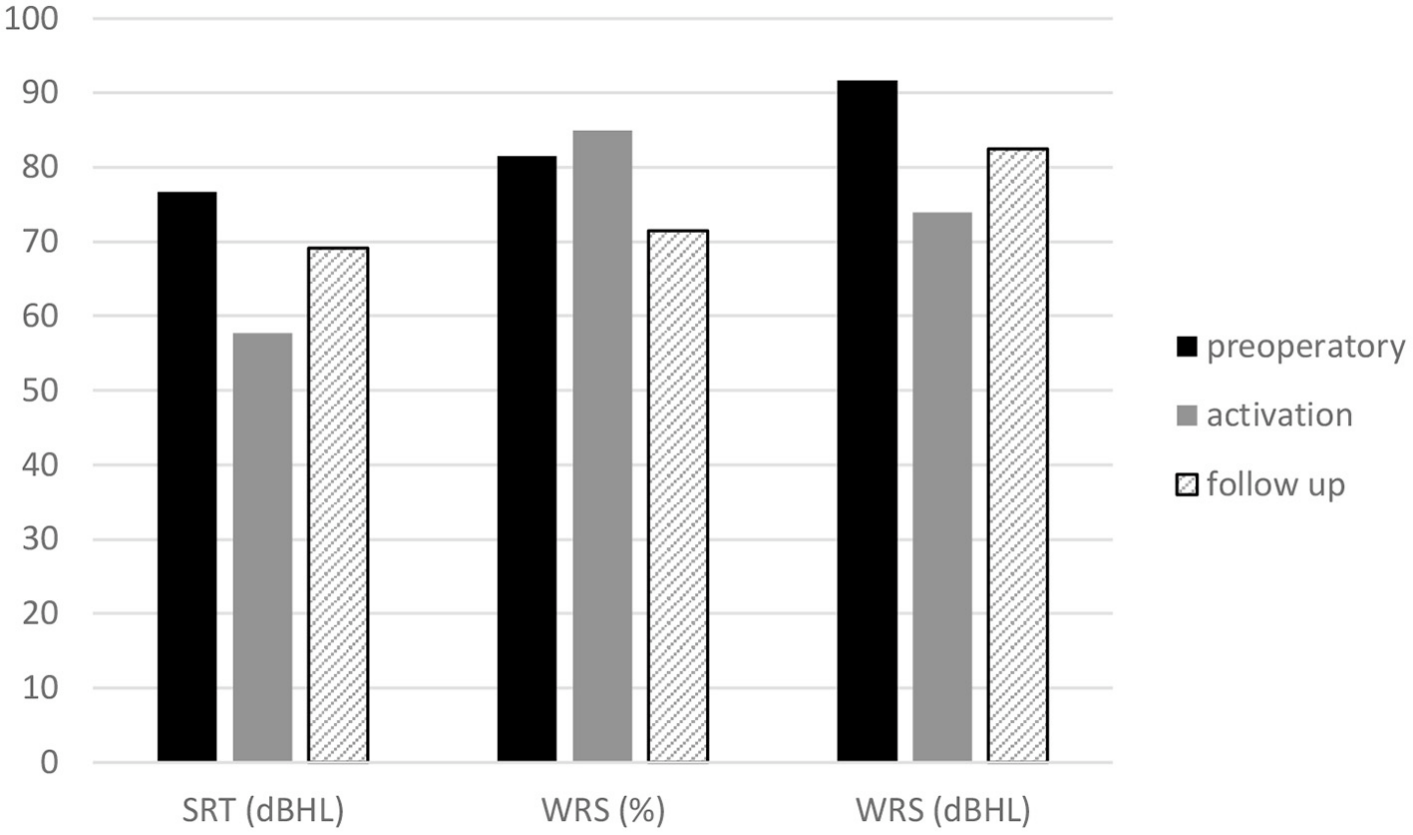
Headphone speech audiometry. Mean speech reception threshold, maximum word recognition score of the Esteem implanted ear at baseline, activation and follow-up.

The mean SRT was 76.75 dB HL preoperatively, 57.75 dB HL at activation, and 69.25 dB HL at the last follow-up. The mean percent WRS was 81.5% preoperatively, 85% at activation, and 71% at the follow-up (Figure 6).

The mean dB HL for the WRS maximum was 91.78 dB HL preoperatively, 74 dB HL at activation, and 82.5 dB HL at the follow-up (Figure 6). The mean SRT, percent WRS and WRS dB HL values that improved at device activation with respect to the preoperative values remained stable at follow-up for SRT and WRS dB HL, while the percent WRS values decreased with respect to activation values (Figure 6). When comparing the SRT and WRS dB HL values of activation with the preoperative values, a significant difference was found in favor of the activation values (*p* =0.0000), while the difference in percent of WRS was not significant. The comparison between the last follow-up and the preoperative speech audiometry values showed significant results only for WRS dB HL in the last follow-up data (*p* = 0.006). The comparison between the last follow-up and the activation speech recognition scores was significant for SRT (*p* = 0.00162) and for WRS dB HL in the last follow-up data (*p* = 0.023).

**Figure 7:**
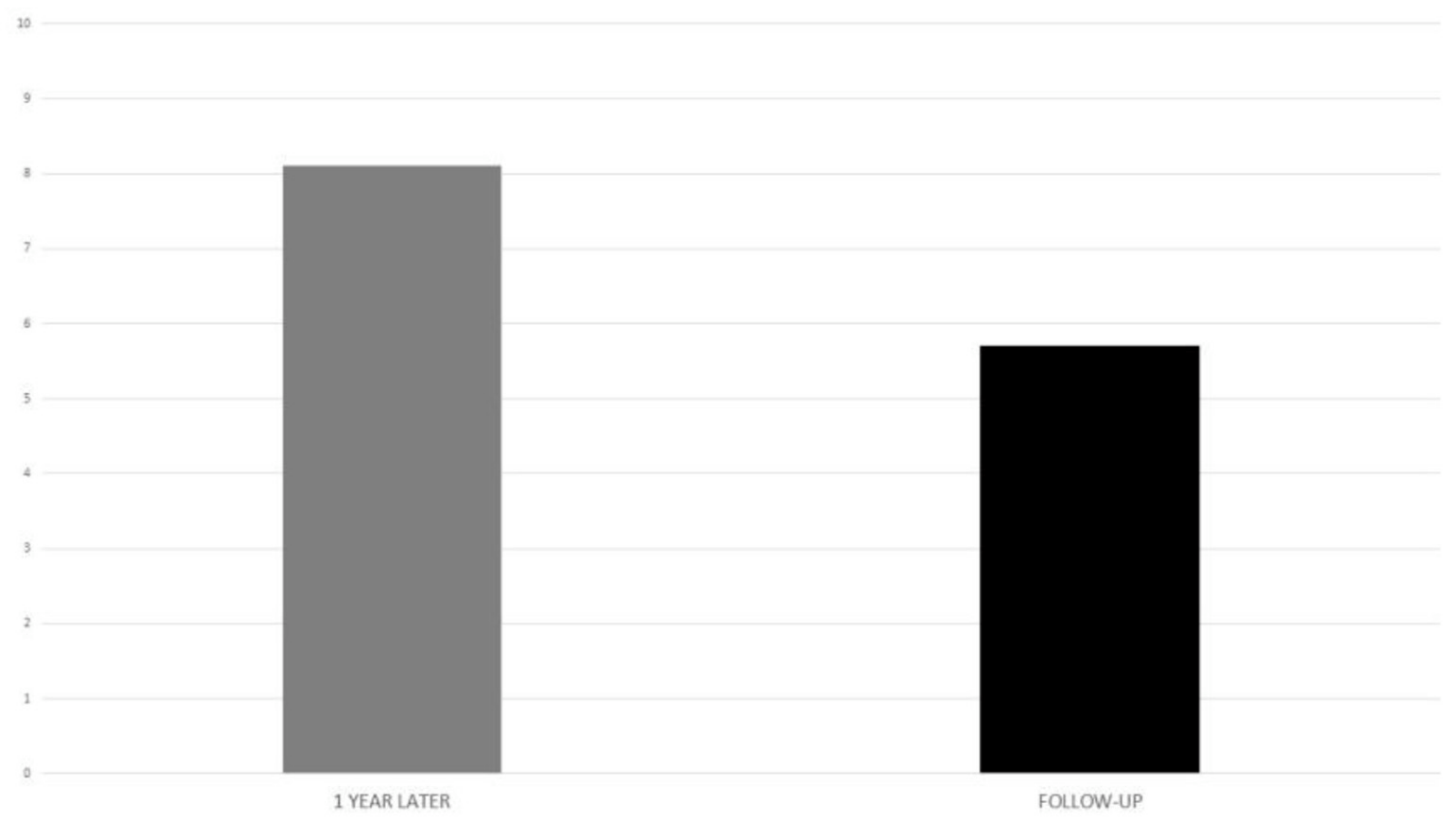
Visual analog scale in the implanted subjects, at activation and follow-up.

Visual analog scale regarding implant benefit, administered at 1 year after implant activation and at the last follow-up, showed a mean value of 8.1 and 5.7, respectively (Figure 7).

### Statistical Analysis (Correlations)

The different timings of follow-up were not significantly correlated with the degree of hearing loss (*p* > 0.05) in either ear (Table 2). The correlation of the degree of hearing loss at the last follow-up to the baseline values was significant in the implanted ear (*p* = 0.02) but not in the contralateral ear (*p* = 0.17) (Table 3).

**Table 2:** Correlation between timing of the follow-up and hearing loss severity.

	**Esteem ear**	**CTR ear**
n. pts	20	20
Correlation coefficient *r*	−0.1035	0.1648
Significance level	*P* = 0.6641	*P* = 0.4876
95% confidence interval for *r*	−0.5221 to 0.3553	−0.2996 to 0.5660

*CTR, Contralateral ear*.

**Table 3:** Correlation of hearing loss grade at follow-up vs. the pre-operative one in the Esteem implanted ear (*p* = 0.0213) and in the contralateral one (*p* = 0.1761).

	**Esteem ear**	**CTR ear**
*N*	20	20
Correlation coefficient *r*	0.5110	0.3150
Significance level	***P*** **=** **0.0213**	*P* = 0.1761
95% confidence interval for *r*	0.08843–0.7777	−0.1481 to 0.6649

*CTR, Contralateral ear. Bold values indicate the statistically-significant data*.

## Discussion

The Esteem® FI-AMEI has previously been reported to be beneficial not only for moderate and moderate-to-severe forms of SNHL (9) but also for more advanced types of hearing loss that cannot benefit from conventional auditory rehabilitation and would be more suitable for cochlear implants (2, 12–14). The Esteem® was well-accepted by patients for its characteristics of bypassing external auditory canal involvement, for its aesthetic qualities, and for providing better amplification in the high-frequency range (3–6 kHz), which is important for speech recognition (15, 16). In a multicenter study that was previously performed to evaluate the safety and functionality of the device during the FDA phase 1 clinical trial (7), the behavior of the BC hearing threshold was reported without considering the symmetry or asymmetry of hearing loss, a parameter that was only taken into consideration for the selection of the ear to be (first) implanted. The 1-year results from the phase 2 clinical trial (9) also highlighted the fact that “*because inclusion criteria did not require symmetric hearing loss, the postoperative hearing with the device was not compared with that of the contralateral aided ear because additional variables would have been introduced*.” This assumption could be explained by the fact that the trial follow-up was limited to only 1 year from surgery.

Since the primary goal of the present study was to monitor the BC threshold over time, using different follow-up periods, the assessment of the hearing threshold in the contralateral, non-implanted ear would appear to be crucial for shedding some light on the causes of eventual BC deterioration. First, in the immediate postoperative period, a change in the auditory pattern in all Esteem-implanted ears regarding the additional conductive component related to the interruption of ossicular chain continuity occurs, which is mandatory for a correct alternate action between the two implanted transducers (7, 17, 18). From a practical point of view, this loss would only impact the eventual use of a CHA in the operated ear, and it will have scarce influence on the functional outcome of the device, which only relies on the BC threshold. In this regard, a report from a phase 2 FDA clinical trial referred to a gradual improvement of the hearing threshold occurring over time of use, with the best fitting shown at 12 months postoperatively in relation to healing and brain training (9).

The present study was designed as a retrospective, non-randomized, single center, clinically controlled study and included 20 Esteem implantees selected on the basis of bilateral symmetric SNHL. The implanted ear was initially chosen considering either an eventual greater degree of hearing loss or the favorable anatomical features of the mastoid size on the preoperative computed tomography (CT) evaluation. By following these patients over several years, it was possible not only to assess the efficacy of the implant after different durations of use but also to monitor the stability of the BC threshold, considering the contralateral ear as a control. The study group was heterogeneous for the severity of baseline hearing loss in the implanted ear since it included subjects who had moderate-to-severe SNHL (group A) and severe-to-profound SNHL (group B), and who had either flat or down-sloping curve shapes. The group was homogeneous for follow-up timing and for the symmetry of the baseline hearing threshold between the two ears in the same subject. Moreover, group B included mostly subjects with a down-sloping baseline auditory shape, which can be thought of as being on the border of an off-label indication, according to the manufacturer's recommendations. As mentioned before, the two groups were homogenous for the variables “follow-up timing” and “severity of the baseline hearing loss” of the implanted ear since no difference for these two variables was statistically demonstrated. Surprisingly, when assessing eventual differences in hearing loss deterioration as a function of the severity of baseline hearing loss, group A (moderate-to-severe) showed a more significant worsening than did group B (severe-to-profound) (*p* = 0.000). Another interesting finding regards the role played by the baseline BC threshold morphology, showing that the flat-shaped curves presented a greater worsening than the down-sloping curves did. Worsening prevailed at 250 and 500 Hz for both curve shapes, without a statistically significant difference with respect to the other frequencies. The explanation for this finding could be related to the necessity of changing the fitting parameters during the follow-up times. In fact, in the case of an auditory decrease at high frequencies, the adopted fitting strategy involved cutting the frequencies between 8 and 12 kHz, considered the upper limit for a standard fitting session, to concentrate more energy toward the lower frequencies that could therefore be more solicited by the piezoelectric mechanical vibration. Another factor could be the minimal impedance improvement created during the surgery, with a bridge between the incus and stapes that should not impact the microphonic role of the tympanic membrane microphone. One important factor is related to the long observation time that allowed us to monitor the BC thresholds of both implanted and non-implanted ears over several years, with differences that did not statistically influence the severity of the final BC thresholds.

When considering the difference in auditory deterioration of the baseline BC threshold in the implanted and contralateral ears of each subject, worsening was recorded in both ears, probably due to the age related hearing impairment—ARHI (19). Even though hearing loss involves both ears, when we statistically compared the implanted ear with the contralateral one, the bone conduction worsening was greater on the implanted side, with a higher significant value for the implanted ear that also showed a positive correlation between the follow-up hearing loss grade and the baseline hearing loss. The different behavior of the implanted ear with respect to the contralateral ear was such that they shifted from being symmetric in the baseline situation to becoming asymmetric at the follow-up. This observation was fundamental to understanding the possible reasons for the greater vulnerability of the implanted ear than that of the contralateral ear, since the initial symmetric levels would assume a univocal origin for hearing loss. Moreover, the asymmetrical hearing loss progression would rule out the role of ARHI, the latter usually involving both ears in a symmetrical fashion.

An important scope of the present study was to observe how stable the FI-AMEI efficacy was over a period of years, regardless of the BC threshold change. In fact, PTA and speech audiometry, as measured at the follow-up controls, reproduced an outcome close to that assessed after the first activation. Therefore, it is possible to state that the performance of the implanted ear remained nearly stable over the years despite the assessed deterioration of the BC threshold. In this regard, the VAS questionnaire showed decreasing satisfaction levels at follow-up as compared to those found at 1 year after device activation, although still positive (score >6), despite the progressive bone conduction impairment. One may therefore assume that, if associated with a customized fitting program, the Esteem® device is able to provide an audiological benefit even in patients with severe hearing loss (20).

The different degrees of deterioration between the two ears may allow us to rule out the importance of the etiology of SNHL. In fact, a certain degree of deterioration was also shown in the non-implanted ear without reaching the low levels shown by the FI-AMEI ear. In this regard, the duration of the follow-up showed no influence, with the same degree of threshold deterioration observed over both the minimum (4 years) and the maximum time (12 years). Likewise, the age of the patients and the severity of hearing loss did not influence the analysis, considering that the study group was formed of subject suffering from moderate-to-severe and severe-to-profound SNHL. In other words, a worse baseline threshold did not result in a greater likelihood of hearing deterioration than a milder threshold did. Therefore, the cause must be considered in other non-pathophysiological factors, such as the energy delivered by the device needing to be progressively increased to adapt to the new demanding situations related to deteriorated BC thresholds. This factor may also be the cause of an early depletion of the battery, which resulted in a mean life of 3.5 years instead of the 5–9 years predicted by the manufacturer (14). Another factor could be inherent in the piezoelectric technology upon which the mechano-electrical function of the two transducers is based. This mechanism depends on certain ceramics or crystals, allowing them to flex when crossed by an electric current or to generate a ZQ current with their deflection, with more energy efficiency than that of the electromagnetic modality (7). Some authors have focused on the negative effects of piezoelectric systems due to the erroneous direction of the expansion of the material, which contracts rather than expands in the direction of the applied electric field (21). From our findings, it can be noticed that the BC deterioration mostly affected the low-mid frequencies and not, as one would expect, the high frequency cochlear range. One may therefore assume that the BC deterioration in the implanted ear could be due to the greater stimulation energy needed for an efficient performance, even in the presence of BC worsening.

It is possible to conclude that the BC threshold in some of the Esteem® implanted subjects can deteriorate over the time, but that its amplification properties could still provide a beneficial role for the implantees, with only a small percentage of them obliged to further rehabilitation with a cochlear implant.

## Data Availability Statement

The raw data supporting the conclusions of this article will be made available by the authors, without undue reservation.

## Ethics Statement

The studies involving human participants were reviewed and approved by Sapienza University. The patients/participants provided their written informed consent to participate in this study.

## Author Contributions

All authors listed have made a substantial, direct, and intellectual contribution to the work and approved it for publication.

## Conflict of Interest

The authors declare that the research was conducted in the absence of any commercial or financial relationships that could be construed as a potential conflict of interest.

## Publisher's Note

All claims expressed in this article are solely those of the authors and do not necessarily represent those of their affiliated organizations, or those of the publisher, the editors and the reviewers. Any product that may be evaluated in this article, or claim that may be made by its manufacturer, is not guaranteed or endorsed by the publisher.
